# President’s Address (November 2016): American Society of Tropical Medicine and Hygiene

**DOI:** 10.4269/ajtmh.17-0389

**Published:** 2017-07-12

**Authors:** Stephen Higgs

**Affiliations:** 1 Biosecurity Research Institute, Pat Roberts Hall, Kansas State University, Manhattan, Kansas

The American Society of Tropical Medicine and Hygiene (ASTMH) has a fascinating and remarkable history. In this Presidential Address, I am going to tell you a story about the Society that I love. I am going to tell the story for the younger people who have become increasingly numerous and influential in our Society, for the international members, and especially for those who are attending our meeting for the first time.

**Figure:**
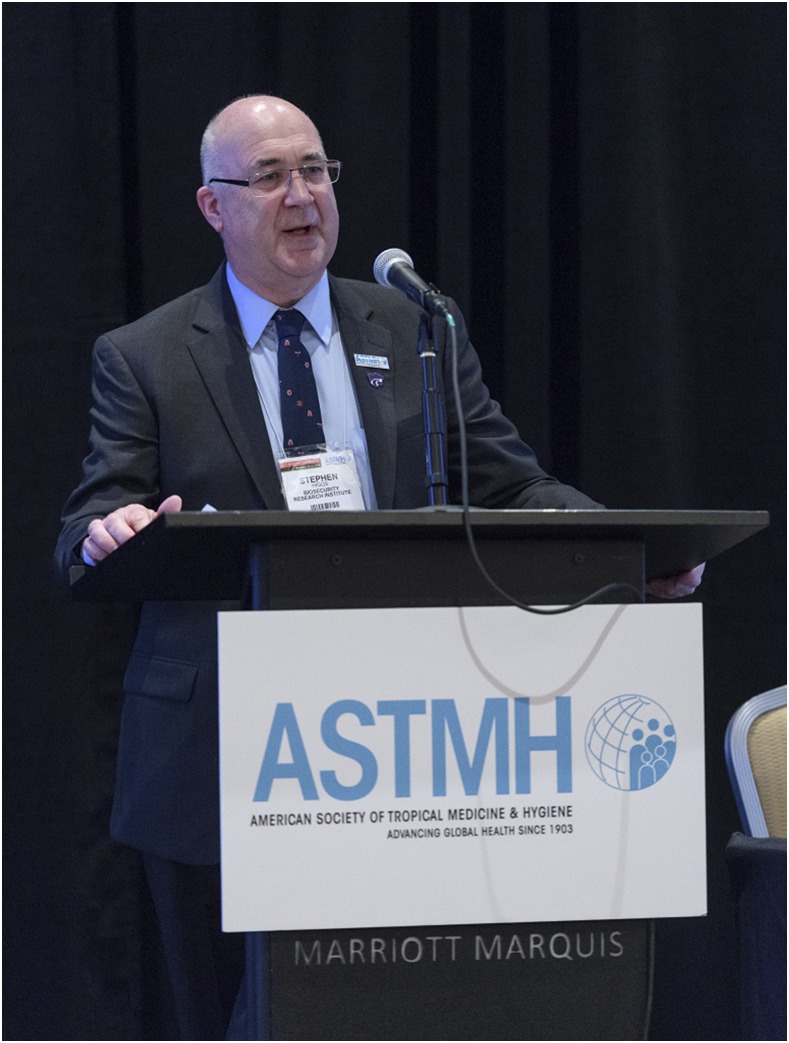
ASTMH President Stephen Higgs, PhD, FRES, FASTMH, gives his Presidential Address at the 2016 Annual Meeting in Atlanta.

A question that people may have asked a long time ago, and perhaps still ask is, Why does the United States need a scientific society focused on tropical medicine and hygiene? The reason is that during the 18th and 19th centuries, infectious tropical diseases were having a tremendous impact on the United States. Yellow fever was the most dreaded disease in the whole of North America, causing approximately 100,000 deaths from multiple outbreaks throughout the United States and the entire continent. I want to thank Past President and Society historian Don Burke, who kindly provided information and many of these slides.

**Figure:**
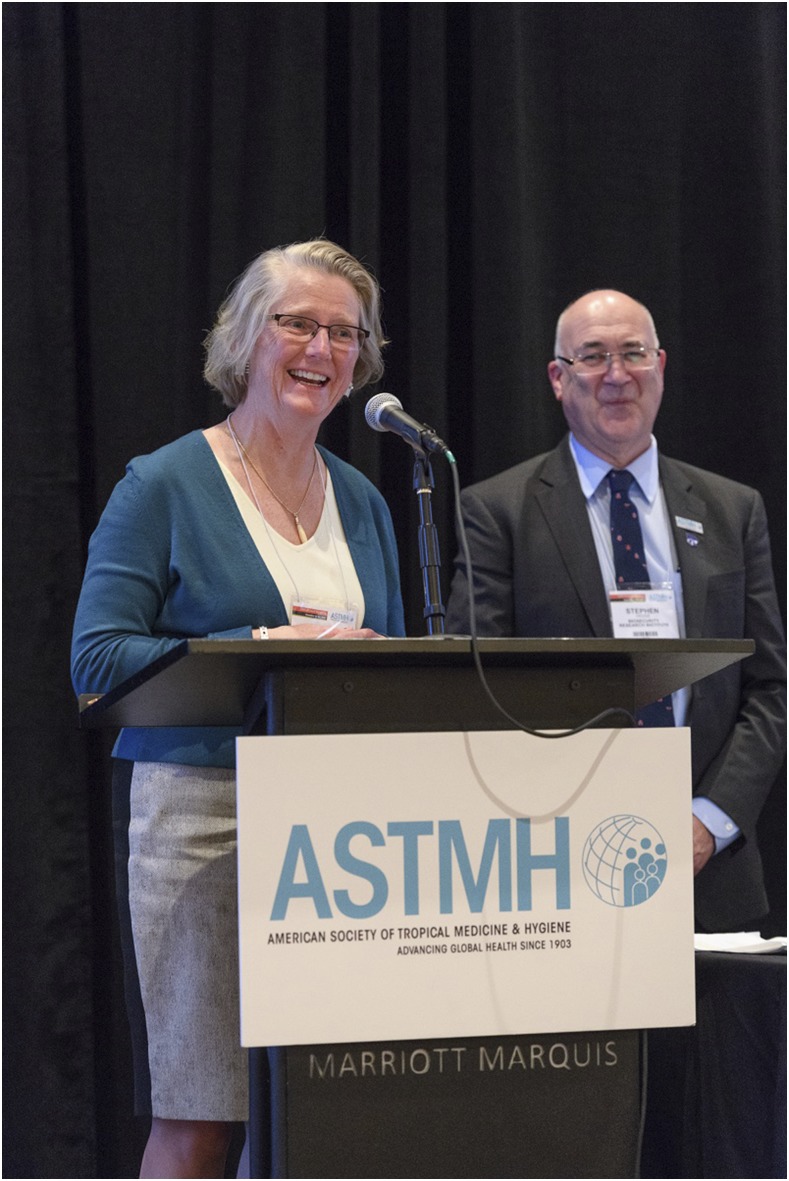
Incoming President Patricia F. Walker, MD, DTM&H, FASTMH (left) speaks during the Business Meeting at the 2016 Annual Meeting in Atlanta while Past President Stephen Higgs, PhD, FRES, FASTMH, looks on.

The cause and transmission cycle of yellow fever was a mystery. A physician, Carlos Finlay, hypothesized that mosquitoes might be involved in the transmission. For the trainee members of our Society, it is important to note that only one of the 104 experiments he conducted actually supported his theory. But that one experiment provided the critical data needed for us to understand how people became infected with yellow fever virus—even before we knew that a virus was responsible for the disease. Repetition is one thing but 104 times? That is tenacity. You have to admire him for his persistence and belief in himself.

**Figure:**
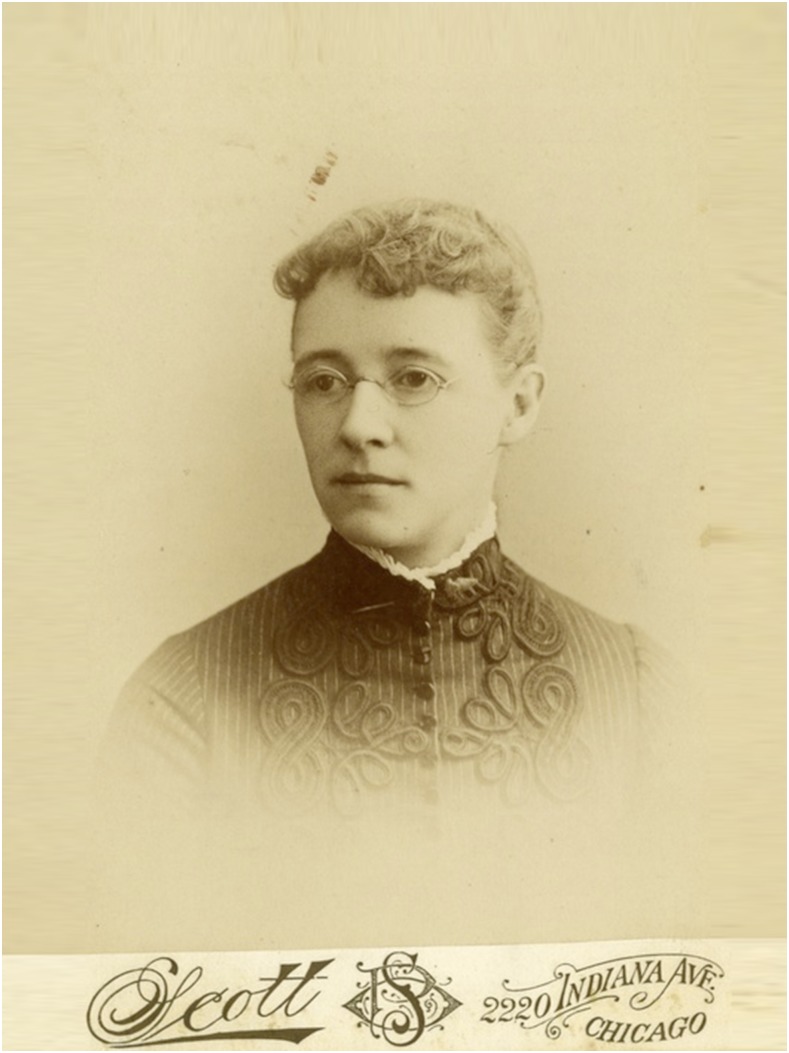
Clara Ludlow (photo courtesy George Washington University Archives).

Tropical diseases also had a very significant impact on the U.S. military. In the Spanish–American War of 1898, approximately a thousand people were killed in combat but there were over 5,000 who died because of disease, mostly due to yellow fever. In response, the U.S. Army developed a Yellow Fever Commission. They performed studies with human volunteers—the sort of thing you could never do today—to prove transmission by mosquitoes, and discovered things that had never really been thought of before; things like the extrinsic incubation period.

**Figure:**
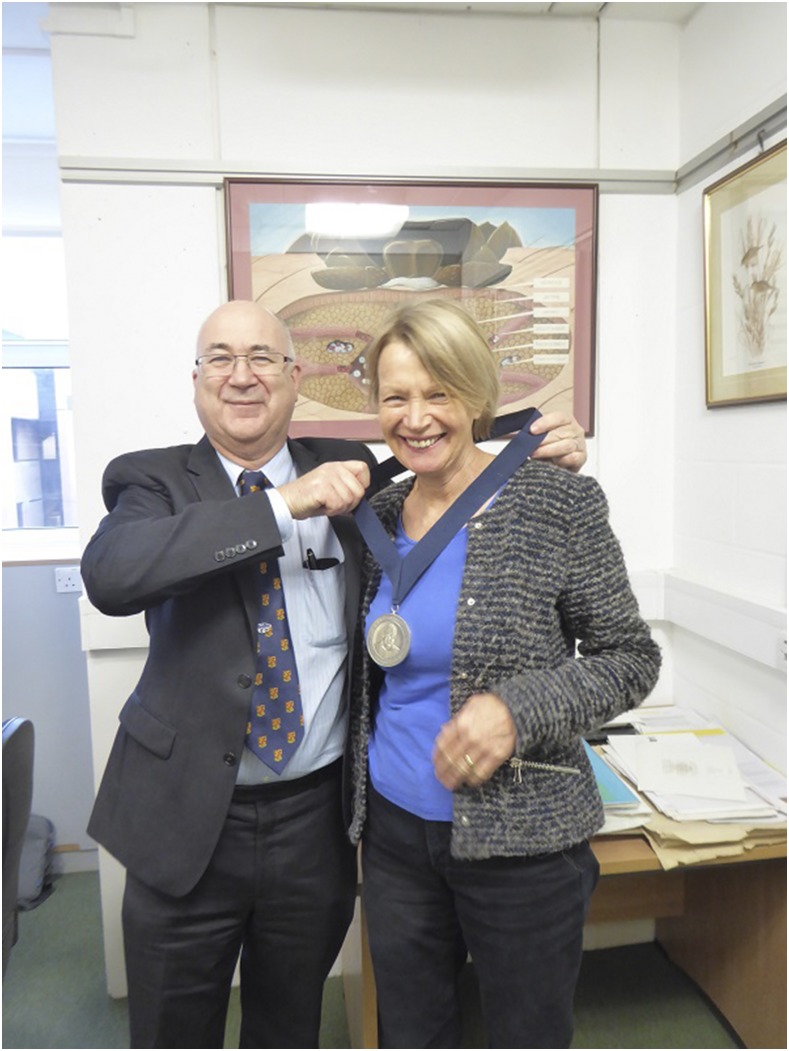
Stephen Higgs, PhD, FRES, FASTMH (left) and 2016 Harry Hoogstraal Medal recipient Patricia Nuttall, BSc, MA, PhD (photo courtesy Stephen Higgs).

Then, of course, there was the impact tropical diseases had on commerce. At the time, construction of the Panama Canal was taking a tremendous death toll on its workers. In a letter to Henry Rose Carter, William Crawford Gorgas wrote that evidence may support the idea that mosquitoes are involved in this disease. When Gorgas’ team went to Panama in 1904 to combat yellow fever, they all contracted malaria; however, within just over a year, thanks to what we now call source reduction, they successfully eliminated yellow fever from Panama.

Malaria was not only in Panama but also, for example, in Savannah, Georgia, about 275 miles from Atlanta, the venue for this meeting. People there applied the lessons learned by Gorgas and developed programs to eliminate mosquito breeding sites, until they finally eliminated malaria from the United States in the early 1950s.

The problem of tropical infectious diseases has not gone away. People said long ago that we were beating infectious disease; however, it still accounts for approximately 25% of all deaths worldwide. Vector-borne diseases alone account for about 1 billion deaths a year, as we have been hearing throughout many talks during the Annual Meeting this week. One of the reasons that these diseases continue to affect people is that global travel has greatly increased. Nearly 2 million people travel aboard commercial airlines every year.

This trend of increasing human infections was seen when chikungunya virus was introduced into the Americas and has been repeated with Zika virus. If you look at the latest data for Zika virus on the Centers for Disease Control and Prevention website, at the beginning of November 2016, there has been almost 4,000 cases of travel-related Zika infections reported in the United States and almost 140 locally transmitted cases.

When you look at these numbers and see what is happening around the world, you will surely appreciate the relevance of the ASTMH and realize that what you, our members, do and the impact that this can have has never been as great as it is right now. I honestly believe that we do not know what is going to come next, but our membership is critical for preparing for whatever it may be.

With respect to the Society, it was formed in 1903 by 28 physicians on March 9 and was originally called the Society of Tropical Medicine of Philadelphia. The name was changed 12 days later to the American Society of Tropical Medicine. It has gone through a few reiterations over time. It was the American Society of Tropical Medicine at the same time as we had the National Malaria Committee, which later became the National Malaria Society. In 1952, the two groups combined to form what we represent now, the ASTMH. The first president was Thomas H. Fenton, a physician. The fourth president was William Crawford Gorgas, mentioned earlier, who fought and eliminated yellow fever in Panama.

A first for the Society—and this was not until 1908—was our first female member, Clara Southmayd Ludlow, who was also the first nonphysician scientist member. Clara graduated with a BS in agriculture, and an MA in botany. Clara received her PhD based on a dissertation describing the mosquitoes of the Philippine Islands, the distribution of certain species, and their relationship to the incidence of certain diseases. Clara is the first woman known to have published extensively on the taxonomy of mosquitoes, and so our first female member was, in fact, an entomologist.

Society membership itself was steadily increasing over the years. It had a surge in membership during World War II, when the nation suddenly realized that U.S. troops were being deployed to areas where they were being threatened by tropical diseases. After the war, membership declined but held at approximately 1,000. We have had many distinguished members over the years including several Nobel Prize winners. These Nobel laureates include Thomas Weller, who was responsible for the discovery of varicella zoster virus (chicken pox) and cell cultures for developing the polio virus vaccine, and attenuation by serial passage for vaccines.

Max Theiler received his Nobel Prize for development of the mouse model and the yellow fever vaccine. Over 400 million doses of yellow fever vaccine have been administered and protected people with almost no untoward effects. Recently, use of this vaccine has played a critical role in fighting the outbreaks in Angola and the Democratic Republic of Congo. Albert Sabin was a member of ASTMH. He is known for discovering orthoreoviruses and developing the live virus for the polio vaccine. Another member, Carleton Gajdusek, discovered the kuru prion.

Today, the Society is the largest international scientific organization of experts dedicated to reducing the worldwide burden of tropical infectious diseases and improving global health.

We have five Subgroups, all very important to the Society: the American Committee on Arthropod-Borne Viruses (ACAV), American Committee of Medical Entomology (ACME), American Committee on Tropical Medicine and Travelers’ Health (Clinical Group), American Committee for Molecular, Cellular and Immunoparasitology (ACCTMTH), and the Global Health Subgroup (ACGH), which is growing. It was formed in 2010 and now is a major group for us.

I became quite involved with ACME in particular, and with ACAV over the years. This involvement has been a mechanism that I want to point out to our young people in the audience because it has allowed me to move through leadership positions, meet people, and become more engaged.

Last year, we conducted what we think might be the first-ever membership survey. We found that less than half of our members are members of Subgroups. I want to encourage you to participate. Students can join as many of these Subgroups as they like for free. Members from low-income and low-middle-income countries are entitled to at least one free Subgroup membership. It is very important that we hear your voices.

The *Journal* has been a vital part of the Society since its beginning. It, too, has gone through a number of reiterations over time. In 1913, we published the *American Journal of Tropical Diseases and Preventive Medicine*. It became *The Journal of Tropical Medicine* in 1921. In 1921, Edna A. Hannibal was the first woman to be listed in our *Journal* as an author on a paper, “The Monillias of the gastro-intestinal tract in relationship to sprue.” She subsequently worked on bubonic plague. In 1922, it was renamed again: *The American Journal of Tropical Medicine*. It merged with the *Journal of the National Malaria Society* in 1952 and that was when it became the *American Journal of Tropical Medicine and Hygiene*.

In terms of the *Journal*, approximately a third of the manuscripts received are from the United States. That means the rest are from multiple countries around the world. It is a truly international journal. In the November 2016 issue, an editorial was published authored by the Executive Committee entitled the “(International) American Society of Tropical Medicine and Hygiene.” It has excellent figures and data that reflect the international nature of our Society.

Over the last 10 years, the Society has become increasingly engaged in terms of policy and advocacy. Some members may be skeptical about the value of this activity, but it is absolutely critical to the Society and to our field. I am a convert. During the mid-year Council meeting, the Council visits the House of Representatives and the Senate to meet with politicians and their staffers to tell them about the Society, offer our expertise as a nonpolitical resource, and discuss the need for sustained funding for tropical diseases.

As a resource for policy makers and, of course, most importantly our members, we have a well-designed and very informative website. The website features statements on the Zika virus, a question-and-answer session, and also notes several Society members as participants in the National Academy’s workshop on Zika earlier in the year, indicating that we are known at the highest levels in the nation for our expertise.

The Society took a strong stand on the delayed U.S. federal funding for Zika efforts. In particular, a letter was sent to the leadership of the U.S. House and Senate with further distribution to every member of Congress. The letter was signed by 26 ASTMH presidents—past, present, and future. We cannot take credit for the $1.1 billion in subsequent funding, but the letter got noticed. Karen Goraleski, ASTMH’s Executive Director, received a personal acknowledgment from Dr. Anthony Fauci, the director of National Institute of Allergy and Infectious Diseases.

From a humble beginning with the Society’s first meeting in a small building in Philadelphia with 28 members, we have grown beyond all expectations. At the 2015 Annual Meeting coincidentally held in Philadelphia, there were 4,083 attendees: 50% were members, 28% were trainees, and 40% were international—testimony to the declaration that we are the “International” American Society of Tropical Medicine and Hygiene. In terms of gender equities, for the attendees, the ratio was about 39% females to 61% males at that particular meeting. There were 169 symposia. If you break it down in terms of presenters for symposia, scientific sessions, and abstracts, it is about equal. Overall, if you take all of those symposia, posters, and abstracts, it was 51% male versus 49% female. The result: critical contributions from all people from all nations.

The number of abstracts has shown a general increase to almost 2,000 this year. Also, the breadth of subject topics has increased. I remember when I attended in 1991, it was a busy meeting. But I look at it now and I look at the number of disciplines that are represented, and it is just amazing.

Going back to vector biology presentations, just briefly, one of the reasons I came to the United States a long time ago was to take part in the John D. and Catherine T. MacArthur Foundation’s Biology of Diseases Vectors course. An article was published in *PLoS Neglected Tropical Diseases* a few years ago looking at the increase in activities related to vector-borne viruses and vector biology. Many of the authors are members of ASTMH. Interestingly, for this article, the activities at our Annual Meeting were used as the standard of measurement. That was the bar by which they measured everything. If you look between 1990 and 2005, the steady increase of interest has made an obvious impact on the field.

The Society has many awards, including the Bailey K. Ashford Medal, Ben Kean Medal, Joseph Augustin LePrince Medal, Donald Mackay Medal, Walter Reed Medal, Honorary International Fellow of ASTMH, Elsevier-ASTMH Clinical Research, Young Investigator, and the ASTMH/Bill & Melinda Gates Foundation Travel Awards. These are Society-level medals, but we also have many awards sponsored by the Subgroups for various accomplishments and achievements. I was frequently on this stage when I was with ACME, presenting the Harry Hoogstraal Award for outstanding achievement in medical entomology. Twenty-five recipients since 1987, all men. Finally in 2016, and deservedly so, Patricia Nuttall became the first female recipient of the Hoogstraal Award.

I was also so pleased to announce during the Awards Ceremony earlier this week that we are establishing the first Society-level medal to be named after a female iconic leader in tropical medicine. The Council voted unanimously to support this. President-Elect Patricia Walker will lead a team to identify the iconic leader whose identification will be based on input from the members. This is very, very significant and I am delighted that we have finally reached this point.

We are a global society. I want to emphasize that. We have many international connections. In 2011, there was what was boldly called the First Annual ASTMH meeting in Peru. Willy Lescano and Dan Bausch were involved with this and, of course, Alan Magill. It really has become an Annual Meeting: this year 225 people from 14 different countries attended. This year it was also a true tribute to the late Alan Magill, who had been so influential in its early formation. This meeting symbolized everything for which he stood.

This year we saw the first ASTMH in Kenya. Again, I hope this is a trend that we are going to see continue. Primarily organized by local people at the sixth KEMRI Annual Scientific and Health Conference, Serap Aksoy, ASTMH Councilor, was also very influential. There were 150 attendees, phenomenal posters, and presentations mainly based on presentations that have been made here at the Annual Meeting. Later this year, we hope to have a first ASTMH meeting in Thailand.

At this week’s Annual Meeting in Georgia, we have had tremendous international participation. The director of Pan American Health Organization (PAHO), Carissa Etienne, gave a very emotional, inspirational talk on the Zika virus from her personal experience. Zulfiqar Bhutta’s presentation on global child survival was just awe-inspiring. It was amazing. He works in the Hospital for Sick Children and Aga Khan University in Karachi, Pakistan. If we look at the geographic distribution of our speakers, it encompassed the globe: South America, Africa, Asia. We have the Charles Franklin Craig Lecture, the emergence of Zika with Albert Ko from the Yale School of Public Health, someone who has been working intimately in Brazil seeing the devastation of this disease.

The Honorary International Fellow of ASTMH is only awarded to non-U.S. citizens. International fellows are from multiple countries and are ambassadors for the Society, motivating young people around the world. One duty of the ASTMH President is to make appointments to key roles for the Society. John Aaskov was appointed and agreed to serve on the selection committee for the Honorary International Fellow of ASTMH. Dr. Aaskov is the first non-U.S. person to serve on this committee.

Fellowships and awards include the Burroughs Wellcome Fund/ASTMH Postdoctoral Fellowship in Tropical Infectious Diseases, Benjamin H. Kean Travel Fellowship in Tropical Medicine, Robert E. Shope International Fellowship in Infectious Diseases, and Centennial Travel Award in Basic Science Tropical Disease Research. If we look at data and statistics related to these awards, we see recipients with an approximately equal gender distribution from around the globe, epitomized in our logo. ASTMH is an inclusive Society. Your vote counts and it is important for members to participate in the leadership. The process is, we have a Nominating Committee—members can nominate themselves or be nominated by other members. This year I appointed Rebecca Rico–Hesse, Hector Gorbea from Puerto Rico, who I assume could not be here because they have 30,000 Zika cases at the moment, and then Tony James, a National Academy of Science member and another person who is passionate about training.

By gender, the ASTMH Council approximates to a 50:50 ratio with representation from different focus areas. Significantly, Abdoulaye Djimde is the first international member on the Council. Hopefully, the Society will have many more international members elected to the Council. It is important to know that any member can be on the Council. It may not be easy, it takes a little time, but it is very worthwhile. A key action item that the Society is taking on is work to determine a student or trainee member of the Council thanks to David Fidock, who is leading a task force that is looking at how to implement this. Of course, this will require a change in the bylaws, but the Society needs a trainee member on Council. The trainee member will have full voting rights.

Well it only took 92 years to get our first female President, but we now have Patricia Walker and Regina Rabinovich two in a row.

Membership is at an all-time high. Middle-income/high-income countries are about 53%. Low- and low-middle income countries are about 12%. Student membership is high, at about 24%. International membership is high, at about 35%. By gender, about 59% are male and 41% are female for those who declared their gender in the surveys. In terms of international distribution, 38% are female and 62% are male compared with 43% and 57% in the United States. The Society has been active since 1903, but we have not been collecting much data. Our history is hard to obtain. We have records but we do not have an appointed historian.

From the membership survey done earlier this year, we had a response rate of only about 10%, which is, unfortunately, pretty much par for the course. We would love more people to respond to these surveys, which are relatively simple. Of the responses we received, 43% were from international members and about 14% were from students. I just have to encourage you to respond. We need to hear you, we will listen, the Council will listen.

In conclusion, based on personal experience, our Society offers extraordinary opportunities for ordinary people. I have traveled a lot through the Society, through my positions, and it has been a real privilege to travel to so many countries where I have met many ASTMH members. It really is empowering seeing different cultures. ASTMH is a Society where members become friends and where you meet your heroes. Our members are so welcoming and so kind in spirit, like one of my personal heroes, Bob Shope. This perhaps rather unfashionable ACAV tie that I am wearing tonight belonged to Bob Shope. When Bob Shope died, his family asked me if I wanted something of his and I was given this tie. I have proudly worn this tie to every Annual Meeting since he died. Bob’s spirit lives on in our Society and this tie reminds me that Bob was a champion and ambassador for the Society and our values. He was tremendously successful but remained humble, and accomplished success by being a genuinely nice person. He loved students, he loved training people, he never rested. We should all learn by Bob’s example. I attribute my success to excellent students and colleagues. Having a collaborative spirit and team work are critical. The importance of what we do transcends politics and personal gain. We are in the business of making lives better and, for some of you, actually saving lives. Make this Society a lifelong commitment, because it will enable you to share your knowledge, share your resources, and work well with others. Behind the scenes, there is a tremendous and dedicated team of management and support staff who will help you bring your dreams to reality.

Finally, I would like to thank ASTMH member Dr. Dana Vanlandingham, an arbovirologist, my wife, my soulmate. When I had, what years before as a young ASTMH member in the audience at the Presidential address, an unimaginable dream—that I would like to be the ASTMH President—Dana was behind me all the way. She and our sons, Nicholas and James, have inspired and empowered me over the years with my career and been understanding of my frequent travel to ASTMH and other meetings. I thank them for their unwavering support and encouragement. The birth of our first son, Christopher, was also inspirational; I remember it so clearly. His untimely death, when he was just 19 weeks old, of Sudden Infant Death Syndrome, is a constant reminder of the fragility of life. Christopher is in my prayers every night, in my thoughts all the time. The tragedy of Christopher’s death is part of my inspiration, passion, and commitment to research and education related to tropical diseases. I look at you in the audience and especially the young members from so many different countries as my extended family, reassured that because of your commitment to the ASTMH, because of what you do, that through this Society, my hopes and ultimate goal of improving lives and saving preventable deaths will be fulfilled.

I thank you most sincerely for giving me the privilege of serving you as the president of this great Society.

